# “Seeing What’s Left”: The Effect of Position of Transparent Windows on Product Evaluation

**DOI:** 10.3390/foods7090151

**Published:** 2018-09-13

**Authors:** Gregory Simmonds, Andy T. Woods, Charles Spence

**Affiliations:** Crossmodal Research Laboratory, Department of Experimental Psychology, University of Oxford, Anna Watts Building, Radcliffe Observatory Quarter Woodstock Road, OX2 6GG Oxford, UK; andytwoods@gmail.com (A.T.W.); charles.spence@psy.ox.ac.uk (C.S.)

**Keywords:** packaging, packaging design, transparent packaging, expected taste, food judgements, position

## Abstract

The position of design elements on product packaging has been shown to exert a measurable impact on consumer perception across a number of different studies and product categories. Design elements previously found to influence the consumer through their positioning on the front of pack include product imagery, brand logos, text-based claims, and basic shapes. However, as yet, no empirical research has focused specifically on the relative position of transparent windows; despite the latter being an increasingly prevalent element of many modern packaging designs. This exploratory online study details an experimental investigation of how manipulating the position of a transparent window on a range of visually-presented, novel packaging designs influences consumer evaluations and judgements of the product seen within. Specifically, 110 participants rated 24 different packaging designs (across four product categories: granola, boxed chocolates, pasta, and lemon mousse; each with six window positions: in one of the four quadrants, the top half, or the bottom half) in a within-participants experimental design. Analyses were conducted using Friedman’s tests and Hochberg procedure-adjusted Wilcoxon Signed-Rank Tests. Window position was found to be a non-trivial element of design, with a general preference for windows on the right-hand side being evidenced. Significantly higher scores for expected product tastiness and design attractiveness were consistently identified across all product categories when windows were positioned on the right- vs. left-hand side of the packaging. Effects on the perception of powerfulness, overall liking, quality, and willingness to purchase were identified, but were inconsistent across the different product categories. Very few effects of window verticality were identified, with expected weight of the product not being significantly influenced by window position. The implications of these findings for academics, designers, and brand managers are discussed, with future research directions highlighted.

## 1. Introduction

Most adults in the western world are typically exposed to, and interact with, product packaging many times over the course of each and every day (see Food Marketing Institute, 2017) [1]. The visual design of product packaging itself warrants serious consideration, given that even subtle changes to the design are capable of influencing the consumers’ product perception (Spence, 2016; Stoll, Baecke, and Kenning, 2008; Velasco, Salgado-Montejo, Marmolejo-Ramos, and Spence, 2014) [2,3,4], product experience (e.g., Velasco et al., 2014) [4], purchase intention (Vilnai-Yavetz and Koren, 2013) [5], product consumption (Argo and White, 2012; Deng and Srinivasan, 2013) [6,7], and various other health-related behaviours (Batra, Strecher, and Keller, 2011; Bialkova and Van Trijp, 2010; Bower, Saadat, and Whitten, 2003) [8,9,10].

If manipulated appropriately, even seemingly minor design elements can elicit strong responses in the mind of the consumer. The relative positions of various design elements on packaging have, for example, been found to influence perceptions of product weight (Deng and Kahn, 2009; Van Rompay, Fransen, and Borgelink, 2014) [11,12], brand and product powerfulness (Dong and Gleim, 2018 [13]; Fenko, de Vries, and Van Rompay, 2018 [14]; Machiels and Orth, 2017 [15]; Sundar and Noseworthy, 2014 [16]), product quality (Machiels and Orth, 2017) [15], product healthfulness (Festila and Chrysochou, 2016) [17], calorific content (Thomas and Gierl, 2017) [18], and overall product liking (Westerman et al., 2013 [19]; see also Velasco, Adams, Petit, & Spence, 2018 [20]). These effects have been found for the position of elements that include: basic shapes, product imagery, text, and brand logos. The previously identified effects of design element position on product perceptions are reviewed in Table 1 (and are discussed further below).

### 1.1. The Effects of Vertical Position

The vertical position of different features of packaging design, relative to the package as a whole, have been found to influence a number of product evaluations. Specifically: (1) on perceptions of product weight, with increased perceived heaviness when design elements (i.e., product imagery) happen to be placed towards the bottom of the pack (see Deng and Kahn, 2009; Van Rompay, Fransen and Borgelink, 2014) [11,12], and (2), on perceptions of brand power, with increased power when design elements (i.e., brand logo) are placed towards the top of the pack (see Dong and Gleim, 2018; Machiels and Orth, 2017; Sundar and Noseworthy, 2014) [13,15,16]. Indeed, these effects do not appear to be exclusive to product packaging, with empirical evidence suggesting vertical position (i.e., height) influences such things as the perceived power of a manager, promotes notions of concept abstraction, and judgements of valence (e.g., Giessner & Schubert, 2011 [21], Judge & Cable, 2004 [22]; see Cian, 2017, for a comprehensive review [23]. In both cases, these effects have been found to mediate positive benefits in other evaluations, such as overall liking of the design and purchase intent. However, both effects are moderated by whether these traits are beneficial for, or congruent with, the product. Therefore, a ‘powerful’ brand would benefit most by positioning design elements towards the top of the pack’s front face (a ‘powerful’ position), and likewise, a ‘less powerful’ brand would benefit most by positioning design features towards the base (a ‘less powerful’ position).

Theoretical accounts suggest both effects are the result of conceptual metaphors, wherein one idea (or ‘conceptual domain’) is related to, or understood in terms of, another (Cian, 2017; see Lakoff and Johnson, 1980, 1999) [23,24,25]. Note how in some cases these conceptual metaphors can be witnessed in other domains of our lives, such as language: for example, with words or phrases like ‘his/her highness’, ‘upper-class’, ‘elevated’, and ‘ascendancy’, which connote height as being an analogue for power (cf. Schubert, 2015 [26]).

### 1.2. The Effects of Horizontal Position

Similarly, manipulating the horizontal position of elements in the visual field has been found to result in various similar effects on consumers. For example, that graphical elements displayed on the right-hand side are perceived as being significantly more appealing, practical, and visually pleasing, and significantly less annoying to consumers (Westerman et al., 2013) [19]. Note, however, that these generally desirable effects were not identified as leading to increased purchase intent or expected tastiness for the respective products.

Theories advanced to explain any effect of laterality tend to revolve around ‘processing fluency’, wherein textual information is recalled more accurately after being shown on the right-hand side of packaging designs, whilst graphical information is best recalled when shown on the left. This is thought to be a result of hemispheric asymmetry in the brain (Rettie and Brewer, 2013) [27], with preferential processing of language-based cues in the left hemisphere, and graphical cues in the right hemisphere, leading this increase in accuracy (see Hellige, 2001) [28]. This effect may also be potentially guided by an attentional bias towards right- (compared to left-) aligned text elements, which, when present on printed advertisement posters, also seems to increase overall product liking (see Janiszewski, 1990) [29]. However, Westerman et al.’s (2013) results are contrary to both accounts: an explanation for this has yet to be proffered [19], with the authors calling for more granular investigation into any theory behind such results (which the present research aims to assist).

### 1.3. Comparing the Effects of Vertical and Horizontal Position

As illustrated in Table 1, this small field of literature has already started to investigate the effect of a relatively broad range of design elements and their positions on a similarly broad range of product evaluations. When considering the differences in standardised effect sizes, and relative mean differences where standardised effect sizes cannot be calculated (note that these data are not presented), it seems that the verticality of design elements presents a slightly stronger effect on a number of evaluations than the laterality of elements does (cf. Deroy et al., 2018) [30]. That is, the decision to position a design element at the base of the pack (rather than the top) seems to have a greater influence on the evaluations of the consumer than the decision to place an element on the right (rather than the left). The only exception to this trend would seem to be for logo position, being optimally presented towards the top of the pack.

However, despite these identified trends, large gaps still exist in our current knowledge in this area. A wide range of product evaluations have been assessed experimentally in this field, yet, this breadth of investigation provides little overlap between studies, and where such overlap does exist, the results can be contradictory, despite conceptual metaphors being used as the theoretical account to support the conclusions of each. Thus, it is unknown how different evaluations interact with each other to lead to general preference and behavioural intentions (e.g., how evaluations of expected tastiness influence the intent to purchase). Furthermore, the possible influence of position on many design elements has yet to be established. For example, to date, no study has assessed whether the relative position of transparent windows is capable of influencing consumers’ judgements, and if so, in what manner. This, despite the fact that transparency is becoming an increasingly prevalent feature on modern packaging designs (see Simmonds and Spence, 2017, for a review) [31], and has a similar, if not exaggerated, effect on consumer’s product perceptions as compared to the use of product imagery (Simmonds, Woods, and Spence, 2018a) [32]. Therefore, the further investigation of the effects elicited on consumers by transparent windows seems warranted.

### 1.4. Research Aims

The present exploratory research has two major aims. First, to identify whether the previously-identified preference for bottom-aligned design elements, and relatively weaker preference for right-aligned design elements, is identifiable for the position of a transparent window. Second, and more broadly, to fill a gap in current knowledge by exploring whether manipulating the position of a transparent window on product packaging influences the consumer, in ways that are similar to those that have been identified previously (albeit for different elements of packaging designs). It seems reasonable, given the current published evidence, to expect that transparent elements should elicit similar effects to those previously identified by manipulating the position of product imagery, since both provide visual information about the product contained within (albeit with varying degrees of reliability; e.g., Chandran, Batra, and Lawrence, 2009) [33]. Exploratory analyses aim to investigate whether this might be the case. The hope is that this research will assist academics, from fields including sensory psychology, consumer behaviour, and visual cognition, in hypothesis generation and validation; will help designers identify ways to optimise packaging design; and will highlight to brand managers how seemingly trivial packaging design decisions can often have a significant and meaningful impact upon consumers’ evaluations and behavioural intentions.

## 2. Materials and Methods 

### 2.1. Participants

One-hundred-and-ten individuals (47 males, 63 females), recruited from Prolific Academic [34], took part in this exploratory experiment in return for a payment of 0.85 GB pounds. The participants ranged in age from 18 to 60 years (Mean (*M*) = 32.7 years, Standard deviation (*SD*) = 10.4). Prolific Academic’s ‘country of origin’ filter was used, meaning that only those individuals who reported having been born in the United Kingdom could decide whether to participate. Seventy participants (63.6%) had bought granola in the past 6 months, 102 had bought boxed chocolates (92.7%), 99 had bought dried pasta (90.0%), and 44 had bought lemon mousse (40.0%). The experiment was conducted on 18 August 2016 between 11:30–13:30 BST (see Woods, Velasco, Levitan, Wan, and Spence, 2015, for a methodological overview of internet-based psychological research) [35], with participants taking an average of 822 s to complete the study (*SD* = 401 s; average payment of £3.72/h). This research was approved by Oxford University’s Medical Sciences Inter-Divisional Research Ethics Committee (approval reference MS-IDREC-R43591/RE001). Each participant provided informed consent prior to taking part in the study.

### 2.2. Stimuli

**Figure 1:**
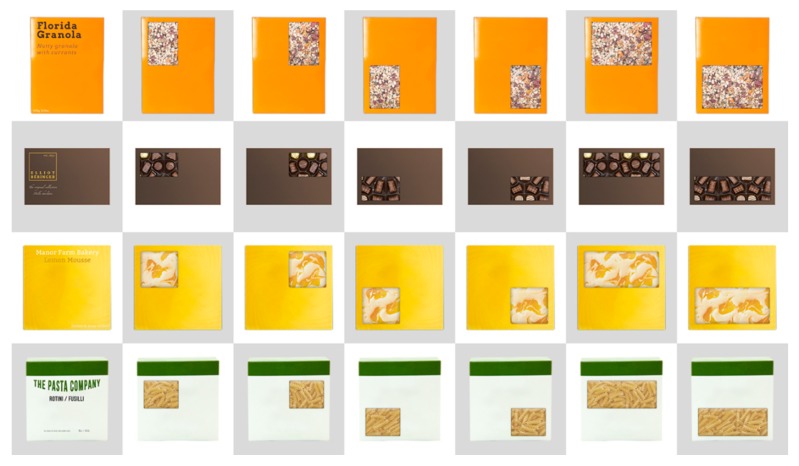
The experimental stimuli shown to participants. The four categories of product (granola, chocolates, lemon mousse, and pasta) are shown with the six possible window positions and sizes (4 quarter- and 2 half-window positions).

The stimuli, research design, and procedure were all similar to those reported in Simmonds, Woods, and Spence (2017) [31]. Four sets of stimuli were created to represent four major food categories: cereal (granola), boxed chocolates, chilled desserts (lemon mousse), and dried pasta. Each design included a transparent window, such that the ‘product’ could be seen clearly. The position of this window varied for each design per product category. The six window positions used were: top-left, top-right, bottom-left, bottom-right, top, and bottom. The former four window positions, which occupied approximately one quarter of the packaging design (‘quarter-windows’), were all of the same size; similarly, the latter two window positions, which occupied approximately half of the packaging’s front façade (‘half-windows’), were both of identical size. Together, these window positions occupied the majority of the visible package face, and as such, no additional information could be included in individual designs for lack of space (given that such information would have needed to be held in a constant position if it were to be included). To ensure that the packaging designs appeared credible, and in order to maximise ecological validity wherever possible, a version of these designs was created that included relevant product information, such as brand name and product heaviness, but with no product window present. These designs were shown to participants prior to testing in order to illustrate what the branding might look like, irrespective of window position. All of the designs were created using brands that do not currently exist in the marketplace (i.e., ‘faux-brands’). All of the stimuli were produced using Adobe Photoshop CS6 software. Those packaging stimuli that were wider than they were tall (i.e., chocolates, pasta, and lemon mousse) were resized to have a fixed width of 200-pixels, while those with packages taller than they were wide (i.e., granola) had a fixed width of 170-pixels (see Figure 1 for the set of stimuli used). These sizes were necessary to permit multiple images to be shown adjacent to one another, without exceeding the minimum screen size (mentioned below).

In order for the participants to be able to complete the study, their monitor needed a resolution that was equal to, or greater than, 1024 × 768-pixels. The experiment was conducted full-screen, thus occupying the entirety of the participant’s monitor. The experiment was displayed in a 1024 × 768 box in the centre of the screen, with whatever remaining space outside the boundaries of this box being occupied by white space. The experiment was conducted online using the Haxe-based Xperiment 2 software compiled into JavaScript (see Haxe Foundation, 2018 [36]; Woods, n.d. [37]).

### 2.3. Design

A 4 × 6 (product category by window position) within-participants experimental design was used, with all stimuli being shown to every participant. Seven questions were asked for each stimulus: overall liking (‘How much do you like the product shown overall?’); WTP (Willingness To Purchase; ‘How likely would you be to buy this product, assuming it was available and at a reasonable price?’); expected tastiness (‘How tasty would you expect this product to be?’); expected quality (‘What quality would you expect this product to be?’); perceived brand powerfulness (‘How powerful would you expect the brand of this product to be?’); expected heaviness (‘How heavy would you expect this product to be?’); and perceived attractiveness of the visual design (‘How attractive is this packaging design to you?’). There was a total of 28 trials. Information was recorded prior to the main experiment concerning the participant’s age, sex, and whether they had bought a product from each of the four product categories that the stimuli were created from in the past 6 months. (Note that this information was used for a segmentation analysis, in order to assess whether any pattern of results significantly different by participant profile. However, no such significantly different segments could be identified across the sample. Purchase frequency was not assessed.)

### 2.4. Procedure

Responses were collected on a roughly 1000 × 350-pixel ‘box scale’ (similar to that used by Van Doorn et al., 2017) [38], on which maximal/minimal responses were anchored (e.g., with ‘Like the product very much’ and ‘Don’t like the product at all’, respectively). The maximal response was always anchored on the right-hand side of the scale. To indicate a response, the participants were instructed to drag the image of the relevant stimulus into the box, where the horizontal position matched how strongly they thought that each stimulus matched the scale presented. The range of possible responses was between 0–100. All six of the stimuli in a product category set were presented above this scale at the same time for each question: there was no limit on presentation duration for the stimuli (i.e., they were visible until all responses were given and the participant chose to proceed to the next screen of the experiment). The stimuli could be placed inside the scale so that they overlapped (the most recently moved stimulus would then occlude any stimuli situated behind it). The height of the box meant that several stimuli could be ‘stacked’ vertically, such that they could be seen simultaneously. The order in which the stimuli were presented above the box scale, as well as the order of faux-brand sets for each question, was randomised (using a robust random-number selection algorithm generated by the online experimental software). Question order was not randomized, and followed the same order as listed previously. The participants could not proceed to the next question until they had provided a response for all stimuli presented above the box scale. After completing the study, the participants were debriefed as to the purpose of the study.

### 2.5. Data Analysis

A Shapiro-Wilk test was used to identify whether the sample came from a normally distributed population. Since the test statistic for all measures was significant, and as confirmed by Q-Q plots, the sample was assumed to come from a non-normal distribution. Consequently, non-parametric tests were adopted where appropriate.

Comparisons between scores for each of the six stimuli were performed using Friedman’s tests separately for each product category and measure. Pairwise, post-hoc comparisons were then performed using a Wilcoxon Signed-Rank Test between all ‘quarter-windows’, and separately, between the two ‘half-windows’. The Hochberg procedure (Hochberg, 1988; see also Huang and Hsu, 2007, for a guide) was adopted to control for multiple comparisons made across product categories for each measure [39,40]. This procedure was chosen to adequately control both Type-I and Type-II errors arising from the relatively large number of comparisons made (see Armstrong, 2014; Ludbrook, 1998) [41,42]. Effect sizes were calculated (see Pallant, 2007; Sullivan and Feinn, 2012) such that the main effects within this study could be directly compared [43,44]; standardised effect sizes were calculated (see Nakagawa and Cuthill, 2007) such that comparison with the extant literature could also be made (as displayed in Table 1) [45].

These data and analyses can be accessed via Mendeley Data (Simmonds, Woods, and Spence, 2018b) [46].

## 3. Results

All Friedman’s Tests returned significant results, with the exception of expected heaviness in the granola, chocolates, and pasta categories. As such, the planned Wilcoxon’s Signed-Ranks Tests were conducted as post-hoc tests on all measures receiving a significant Friedman’s Test score, testing the six permutations between all ‘quarter-window’ stimuli (150 comparisons). Additionally, for the ‘half-window’, Wilcoxon’s Signed-Ranks Tests were used to test differences in the same manner (28 comparisons).

### 3.1. Descriptive Statistics

Table 2 and Table 3 show descriptive statistics for each measure across all stimuli: Table 2, for the quarter-window stimulus set, and Table 3 for the half-window stimulus set.

The quarter-window results highlight that both the top-right and bottom-right positions consistently received higher scores against the top-left and bottom-left positions across the majority of measures, and for all categories. Indeed, at least one of these two rightward positions always received the highest score on every measure across every category. In no instance was the score between both of the rightward positions, nor for between both of the leftward positions, significantly different. However, scores across the majority of measures for stimuli with windows placed towards the bottom of the designs were consistently marginally (but not statistically significantly) greater than those with windows placed towards the top. Similarly, descriptive statistics for the half-window stimuli also suggest a broad (but often non-significant) preference for windows placed at the bottom of the design.

**Figure 2:**
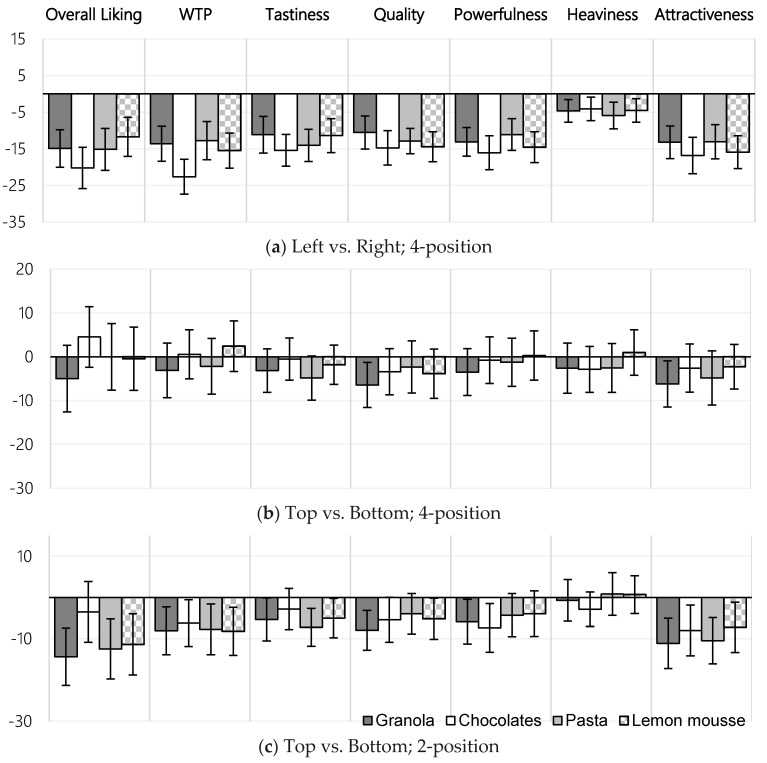
Mean difference between window positions, by measure, clustered by product category. (**a**) The average effect of horizontal window position (average score for left-aligned windows, minus average score for right-aligned windows; i.e., a negative difference shows a higher average score from the stimuli with right-aligned windows), using mean differences from the 4-position stimulus set; (**b**) the average effect of vertical position (average score for top-aligned windows, minus average score for bottom-aligned windows; i.e., a negative difference shows a higher average score from the stimuli with bottom-aligned windows), using mean differences from the 4-position stimulus set; and (**c**) the average effect of vertical position (as above) using mean differences from the 2-position stimulus set. Error bars show the 95% confidence interval about the mean.

Figure 2 shows the average mean difference between window positions across all measures; Figure 2A showing the difference between lateral positions (average score for both leftward windows minus average score for rightward windows) for the four quarter-window stimuli, Figure 2B showing the difference between vertical positions (as before, with top vs. bottom) for the four quarter-window stimuli, and Figure 2C showing the difference between vertical positions for the two half-window stimuli.

Three further trends can be tentatively identified from this graphic analysis. First, that there is a stronger effect of window laterality than there is of verticality for all measures, with windows positioned towards the right more strongly preferred than those positioned towards bottom. Second, that the effect of window position seems relatively uniform across all measures, bar perceived product heaviness. Third, that the effect of window position also seems to vary little between all product types tested.

### 3.2. Inferential Statistics

Findings from the post-hoc Wilcoxon’s Signed-Ranks Tests are shown in Table 2 and Table 3.

#### 3.2.1. Expected Heaviness

No significant main effect was identified for expected heaviness between any window position in any of the four product categories tested.

#### 3.2.2. Perceived Brand Power

A main effect of position on perceived brand powerfulness was identified between lateral window positions. Specifically, between bottom-right and bottom-left positions only in the pasta and lemon mousse categories, and between the bottom-right and both (1) the bottom-left and (2) top-left positions in the granola and chocolates categories. In each of these cases, the rightward window achieved the significantly higher score. However, no significant effect of verticality was identified.

#### 3.2.3. Other Measures

Across all four product categories, WTP, expected tastiness, expected quality, and perceived attractiveness had at least one significantly higher score for a rightward window position, as compared to a leftward position. That is, packaging designs with a transparent window placed on the right-hand side of the design were more likely to be considered for purchase, were expected to contain tastier and better quality products, and were thought of as being more attractive.

Results for overall liking identified a significant main effect of window laterality (with rightward windows resulting in greater overall preference) in all product categories with the exception of the lemon mousse, where no main effect was identified. Designs with windows positioned on the right were liked more overall in every case.

#### 3.2.4. Effect Sizes

Overall liking and WTP had variable effect sizes, ranging from weak (0.33 and 0.32, respectively) to strong (0.72 and 0.74). Expected tastiness and perceived attractiveness had weak to moderate effect sizes (0.35 and 0.32, to 0.55 and 0.59, respectively). Perceived brand powerfulness had consistently moderate effect sizes (0.40 to 0.58). See Appendix A for detailed effect sizes. Note here that such effect sizes are comparable to those identified in prior research that manipulated the position of product imagery (see Table 1). However, note that the effect on overall liking, which was found to be very weak in Westerman et al.’s (2012) study (where the position of basic shapes was manipulated on the lateral axis) [19], was considerably stronger in the present study. This tentatively points to the lateral position of transparent windows being more influential on the evaluations of the consumer than the position of basic shapes.

## 4. Discussion and Conclusions

The results for the effect of window verticality on perceptions of heaviness demonstrates a failure for transparent windows to replicate the results of Deng and Khan (2009) and Van Rompay, Fransen, and Borgelink (2014), where expected heaviness was greater with packaging designs featuring lower windows [11,12]. This is contrary to expectations that a transparent window would elicit similar effects as those identified by manipulating a product image (as both of the aforementioned studies did) as a result of conceptual metaphors involving verticality.

Similarly, a main effect of perceived powerfulness is also not identified when window verticality is manipulated, in contrast with Machiels and Orth (2017) and Sundar and Noseworthy (2014), where elements featured at the top of the design promoted higher perceptions of brand power [15,16]. Again, this result runs counter to the predictions that a conceptual metaphor (or metaphors) would result in higher window positions also increasing perceived powerfulness―though note that prior research had manipulated the position of a brand logo, not a product image.

Note also that previous findings have identified greater purchase intent for products that feature a product image at the top of the design in both Sundar and Noseworthy (2014) and Van Rompay, Fransen, and Borgelink (2014) [12,16]; and at the bottom in Fenko, de Vries, and Van Rompay (2018) [14]. In contrast, the present results identified no main effect of window verticality―instead, finding a main effect of laterality―and highlight further differences in the effects elicited by transparent windows and product imagery.

Finally, a main effect of window verticality is identified for perceptions of overall liking, with more favourable ratings for designs that featured a window on the right-hand side. This corroborates findings of a general preference for rightward graphical elements, as identified previously by Westerman et al. (2013) and Deng and Khan (2009) [11,19].

In sum, the present exploratory research provides several key insights to add to, and help synthesise, the extant literature. First and foremost, that the effect of window laterality (with windows on the right) would appear to elicit stronger and more positive product evaluations than that of window verticality (with windows at the bottom). This is a novel, perhaps unexpected finding, based on prior results: as in Table 1, effect sizes (where calculable) and standardised mean scores (in the absence of effect sizes; data not shown), for bottom- vs. top-aligned windows exceed those for right- vs. left-aligned windows. Conceptual metaphors, which up until now have been used to explain the host of evaluative phenomena resulting from design element position, seem unable to account for the effects identified using transparent windows.

As discussed previously, the presence of prior conflicting results suggests the presence of some boundary condition(s), or otherwise, an unexplored covariate or confounding variable, which has yet to be identified. This research adds to such diversity in existing results by identifying an unexpected effect of laterality. Thus, for any actionable insights to be garnered, a much wider-reaching set of experiments that cover a broad range of design elements, in a wide range of positions on-pack, would be necessary. Indeed, this would seem especially pertinent now that it may be that conceptual metaphors are unable to provide a comprehensive explanation of these phenomena, and that the current state of the literature still does not seem to lend itself to a singular, unified explanation of the cognitive mechanisms behind the effects identified. Future research and theory should investigate these major issues further in order to gain the necessary understanding to explain the effect of design element position on consumer evaluations.

Nevertheless, despite the current lack of theoretical clarity, some direction for future research can be drawn. As suggested by Westerman et al. (2013) [19], perhaps consumers have a general preference for right-aligned graphical (vs. textual) elements, leaving the left-hand side free for quick and less effortful identification of those (textual) product attributes needed to consider purchase. Indeed, it may be the case that a ‘halo-effect’ is positively moderating all variables measured, and across all product categories tested, where windows meet this general rightward preference (and perhaps a weaker bottom-align preference). Given that this pattern was identified in the present research across several product categories for many measures, we suggest this might be generalisable across other product categories.

Second, the present research identifies that varying the position of transparent windows fails to replicate previously identified effects of expected product heaviness and perceived brand powerfulness. That is, we might have expected to see windows positioned at the top of the design eliciting significantly greater expectations of brand power (a result of a conceptual metaphor linking height with power) and a lower window to elicit significantly greater expectations of product heaviness (again, through a conceptual metaphor, in this case linking lower relative position with weight). However, no significant effects were identified in this regard. Note that this may, perhaps, be due to a required specificity of the conceptual metaphor: for example, that perceived brand power cannot be manipulated with conceptual metaphors involving the product; or that heaviness is only manipulated by a graphic printed on pack, rather than a window cut out of the pack. Adding such conditionality seems contrary to the concept of conceptual metaphors, in that they tend to be broad and universal in nature (e.g., ‘high’ connotes ‘powerful’, ‘low’ connotes ‘heavy’). However, one might reasonably expect an image of the product, and a view of the product through a transparent window, to elicit the same conceptual metaphors (since both presumably relate to the product inside), though intriguingly this was found not to be the case here.

### 4.1. Limitations

It is important to highlight a number of limitations of this study. Most importantly, the authors note that the present results have lower than usual statistical power due to a large number of comparisons (across designs, categories, and measures), within-participants manipulations, and a moderate sample size. However, this research was designed to be exploratory in nature, such that a broader understanding of design element position could be garnered. Given the relative consistency in the pattern of results (e.g., the similarity in results between each product category, and even between each variable measured), reassurance is provided that the results are not in part due to ‘false-positive’ statistical inference (and also in accordance to the guidelines established by Simmons, Nelson, and Simonsohn, 2011 [47]; and appropriate for the purpose, as per the advice provided by Stebbins, 2001 [48], cf. Wagenmakers, Wetzels, Borsboom, van der Maas, & Kievit, 2012 [49]). Future researchers are advised to expand with more granular research of the themes identified, using larger base sizes and between-participant designs, such that the statistical power would be greater. Indeed, the use of non-hypothetical research methods, such as real-choice or experimental auction, would help increase ecological validity, and thus, the confidence that could be placed in any recommendations made. Such research might then allow researchers to more confidently make recommendations to commercial stakeholders.

Note also that this experiment was conducted entirely online. While this may be less of an issue in the coming years, given that online grocery shopping is quickly becoming a major channel for grocery purchase (see International Food Information Council Federation, 2018 [50]; Omar, 2005 [51]; Seitz, Pokrivčák, Tóth, and Plevný, 2017 [52]; The Institute of Grocery Distribution, 2017 [53]), it might limit generalisability to physical packaging designs as one would find in the supermarket aisles. Furthermore, since any experience of the product during the experiment was limited solely to the packaging’s visual information (i.e., other sensory information regarding the product or packaging was not available), it is important to note that our results relate only to product expectation, not experience. As such, the results may have less validity when trying to extrapolate them to an in-store setting.

Finally, we feel it important to highlight that the position of only one design element was manipulated: in this case, only transparent windows. This poses a number of challenges: for example, packaging designs are legally required (if not for reasons of aesthetics, as well) to contain much more than just a transparent window in many countries. In the European Union, packaging legislation demands at least the product’s name, nutritional information, weight, and use by/best before date are visible on the primary façade of almost all packaged foods and drinks (see United Kingdom (UK) Government, n.d.) [54]. Thus, in reality, the position of many other elements may also be of tangible influence on the evaluations of the consumer, limiting the external validity of any findings and recommendations. Indeed, given that effects seem to differ by design element (e.g., the effects of heaviness perceptions currently seem specific to image position, and not window position; further, the overall liking of brand logo is preferred at the top of the pack, whereas windows seem preferred towards the right), it would be naïve to expect that these effects exist in isolation. As an additional challenge, current results can provide incompatible recommendations. For example, that a snack food produced by a less-powerful brand would have both brand logo and product image optimally placed at the bottom: no further guidance is offered as to how best to design around such principles. It would certainly be valuable for future research to investigate whether any interaction between design elements and their positions can be found, and, if so, whether the position of any given element takes ‘precedence’ in the minds of consumers.

### 4.2. Recommendations

In light of the fact that this exploratory research lacks the statistical power to make concrete recommendations, several previous recommendations may be adjusted and combined. For example, since it may be that transparent windows cannot influence perceptions of product heaviness, a printed image may be more beneficial in those cases where product weight is an especially important attribute to convey (for example, diet-related products, where the concept of lightness is more important). Furthermore, while current advice recommends that a powerful (i.e., market-dominant) brand should optimally place their logo at the top of their packaging, it may be that adding a rightward transparent window in conjunction would further optimise the design’s impact on favourable evaluations. In addition, most generally, the present results make it seem advisable to place transparent windows on the right-hand side, and keep textual (product- and brand-specific, at least) text to the left. In all cases, these recommendations are only relevant if the addition of a window is practical, not cost-prohibitive, and where the product inside is attractive (as per Simmonds and Spence, 2017; Simmonds, Woods, and Spence, 2018a) [31,32].

### 4.3. Concluding Remarks

The results of the present study provide packaging designers with a statistically robust rule of thumb regarding the optimal position of a transparent window being best placed at the right, bottom, or bottom-right of the package. Such research provides valuable insight into optimal design, and helps to demonstrate the tangible benefit of very cost-effective (each participant costing about 4 pounds Sterling), very rapid (2 h to collect data from 110 people), online research to the packaging industry. Indeed, brand managers and packaging designers would be well advised to research the effect of proposed packaging designs on consumers in order to optimise potential market success, given the difference in effect sizes between product categories, and the unique effects expected to be evoked by different design elements themselves.

## Figures and Tables

**Table 1:** A review of the effects of position on packaging design for different design elements on commonly-reported behavioural and evaluative measures.

Position	Basic Shape	Imagery	Logo	Transparent Window
Top-left	-	He ^(A)^, OL ^(A)^; He ^(D)^, PI_(0.60)_^(D)^	-	-
Top-centre	-	He ^(A)^, OL ^(A)^; PI ^(E)^, In ^(E)^	Po ^(C)^, PI ^(C)^, Po_(0.35)_^(F)^	-
Top-right	-	He ^(D)^, PI ^(D)^	-	-
Middle-left	OL ^(B)^	He ^(A)^, OL ^(A)^	-	-
Middle-centre	-	-	-	-
Middle-right	OL_(0.17)_^(B)^	He ^(A)^, OL ^(A)^	-	-
Bottom-left	-	He ^(D)^, PI ^(D)^	-	-
Bottom-centre	-	He ^(A)^, OL ^(A)^; PI_(0.65)_^(E)^, In_(0.40)_^(E)^	Po ^(C)^, PI ^(C)^, Po ^(F)^	-
Bottom-right	-	He ^(A)^, OL ^(A)^; He_(0.50)_^(D)^, PI ^(D)^	-	-

Key: Each cell in this table shows measures used (see ‘Measures’ below; measures are grouped by reference) for a specific design element (see columns) and position on the packaging design (see rows) across the extant literature (see ‘References’ below). Where a significant main effect of a measure was reported between different positions, the measure is shown in bold for position(s) where the score was highest, with the standardised effect size (Cohen’s d) in subscript (if enough data has been presented to calculate effect size), and with the reference letter (as in ‘References’ below) in superscript. References: **^(A)^** Deng and Kahn (2009) [11]; **^(B)^** Westerman et al. (2013) [19]; **^(C)^** Sundar and Noseworthy (2014) [16]; **^(D)^** Van Rompay, Fransen and Borgelink (2014) [12]; **^(E)^** Fenko, de Vries, and Van Rompay (2018) [14]; **^(F)^** Machiels and Orth (2017) [15]. Measures: He: Perceived product heaviness; In: Expected product intensity (e.g., taste intensity, smell intensity, alcohol content); OL: Overall liking or generalised design appeal; PI: Purchase intent; Po: Perceived product or brand powerfulness; Va: Expected product valence (e.g., tastiness). Note: Measures selected for this review were reported in two or more of the publications listed; other measures (i.e., if reported in only one publication, e.g., expected sale price) are not shown. The following null effects were identified (using the following notation: reference letter (measure 1_(Cohen’s d)_, measure 2_(Cohen’s d)_, …): B(PI, Ta); C(In, OL); D(OL_(0.16)_, Ta, In); E(In_(0.14)_, PI_(0.16)_). Example of how to read table: As reported in ‘Reference A’ (Deng and Khan, 2009 [11]; see any measure with a superscript ‘A’), products were perceived to be significantly heavier (see ‘He’ measures, noting that ‘heavier’ positions are presented in bold) when an image of the product was positioned in the bottom-, right-, or bottom-right positions, as compared to when positioned in the top-, left-, or top-left positions. No effect sizes could be calculated using the data presented by the authors (the table reflects this by omitting the subscript section showing effect size).

**Table 2:** Results for the ‘quarter-window’ (top-left/top-right/bottom-left/bottom-right) stimuli by category.

	Top-Left (TL)		Top-Right (TR)		Bottom-Left (BL)		Bottom-Right (BR)	
	*M*	*SD*	*M*	*SD*	*M*	*SD*	*M*	*SD*
Granola								
Overall liking	43.2	31.1	**56.2 ^TL^**	29.6	46.3	27.0	**63.1 ^TL, BL^**	29.4
WTP	47.0	27.6	**57.3 ^TL^**	29.8	46.8	25.0	**63.7 ^TL, BL^**	27.2
Tastiness	47.3	28.1	**58.0 ^TL^**	29.0	50.0	27.9	**61.6 ^TL, BL^**	27.4
Quality	48.8	25.3	**57.2**	28.3	53.1	25.8	**65.7 ^TL, BL^**	23.4
Powerfulness	41.6	26.3	**56.0 ^TL^**	28.6	46.4	26.3	**58.2 ^TL, BL^**	26.6
*Heaviness*	*47.0*	*24.8*	***52.5***	*28.2*	*50.4*	*24.1*	***54.3***	*26.1*
Attractiveness	37.4	27.4	**51.6 ^TL^**	30.7	44.6	25.7	**56.8 ^TL, BL^**	29.6
Chocolates								
Overall liking	49.5	32.1	**63.2 ^BL^**	31.3	38.4	29.5	**65.2 ^BL^**	27.8
WTP	46.1	27.6	**68.5 ^TL, BL^**	27.3	45.3	27.9	**68.2 ^TL, BL^**	23.5
Tastiness	58.2	28.7	**73.9 ^TL, BL^**	23.4	59.0	29.0	**74.1 ^TL, BL^**	25.7
Quality	54.3	26.7	**67.8 ^TL^**	26.5	56.4	29.9	**72.5 ^TL, BL^**	23.6
Powerfulness	48.7	25.6	**63.3 ^TL^**	25.3	48.0	26.9	**65.6 ^TL, BL^**	26.5
*Heaviness*	*39.9*	*27.8*	***42.2***	*27.3*	*41.0*	*25.8*	***46.9***	*28.9*
Attractiveness	46.7	28.3	**61.6 ^TL^**	28.3	47.3	28.0	**66.1 ^TL, BL^**	29.0
Pasta								
Overall liking	49.3	29.6	**56.9**	32.4	41.8	30.5	**64.5 ^BL^**	28.6
WTP	50.7	29.1	**62.0**	26.6	51.4	30.1	**65.7 ^BL^**	27.0
Tastiness	50.4	28.0	**62.5 ^TL^**	26.7	53.2	29.6	**69.3 ^TL, BL^**	25.1
Quality	50.5	26.9	**59.9 ^TL^**	25.1	49.3	27.0	**65.7 ^TL, BL^**	25.2
Powerfulness	44.1	24.6	**52.5**	27.3	42.6	26.1	**56.5 ^BL^**	27.6
*Heaviness*	*42.6*	*26.8*	***49.3***	*27.3*	*45.9*	*26.9*	***51.0***	*26.7*
Attractiveness	40.5	28.2	**50.0 ^TL^**	30.1	41.8	28.6	**58.4 ^TL, BL^**	29.3
Lemon mousse								
Overall liking	46.1	27.7	**59.5**	32.1	48.2	28.2	**58.3**	32.1
WTP	47.7	24.9	**65.4 ^TL, BL^**	26.7	47.4	26.3	**60.7 ^BL^**	29.7
Tastiness	51.9	27.6	**67.6 ^TL^**	27.4	58.1	28.4	**65.1 ^TL^**	27.1
Quality	47.4	27.8	**65.8 ^TL^**	25.6	55.2	26.6	**65.7 ^TL, BL^**	26.3
Powerfulness	46.6	26.3	**63.5 ^TL, BL^**	26.0	48.6	24.1	**60.9 ^BL^**	24.8
Heaviness	42.2	26.1	**49.4**	29.3	43.9	25.4	**45.7**	27.0
Attractiveness	43.5	29.3	**62.6 ^TL, BL^**	28.6	49.0	27.5	**61.7 ^TL, BL^**	28.4

Superscript letters denote a significantly higher score between measures, where the letters refer to the abbreviated position of the window, and as calculated by post-hoc Wilcoxon Signed-Rank Tests adjusted by the Hochberg procedure. *M* = Mean, *SD* = Standard deviation, WTP = Willingness to Purchase. Note: Sample size = 110. Italicised rows denote cases where the Friedman’s test score was non-significant, and thus, post-hoc significance testing between positions was not performed. The mean score for any window position, on any measure, with a score that is significantly higher than another position, is displayed in bold.

**Table 3:** Results for the ‘half-window’ (top/bottom) stimuli by category.

	Top		Bottom					
	*M*	*SD*	*M*	*SD*	*z*	*r*	*p*	Sig.
Granola								
Overall liking	47.4	28.0	61.8 *	25.8	−3.76	−0.25	0.0002	**Yes**
WTP	50.3	27.8	58.3	28.0	−2.64	−0.18	0.0083	No
Tastiness	51.9	27.9	57.2	29.0	−2.13	−0.14	0.0328	No
Quality	54.0	24.3	62.0 *	25.3	−3.63	−0.24	0.0003	**Yes**
Powerfulness	48.9	25.1	54.8	25.7	−2.73	−0.18	0.0064	No
Heaviness	56.5	23.3	57.2	25.7	−0.36	−0.02	0.7217	No
Attractiveness	46.4	26.6	57.5 *	26.9	−3.63	−0.24	0.0003	**Yes**
Chocolates								
Overall liking	59.8	27.5	63.3	27.3	−1.01	−0.07	0.3126	No
WTP	58.8	27.4	65.0	27.1	−2.41	−0.16	0.0161	No
Tastiness	68.5	25.5	71.3	24.3	−0.78	−0.05	0.4381	No
Quality	65.1	25.8	70.5	23.7	−2.13	−0.14	0.0333	No
Powerfulness	56.0	25.5	63.4	26.2	−2.22	−0.15	0.0263	No
Heaviness	45.1	26.4	48.0	28.0	−1.70	−0.11	0.0884	No
Attractiveness	59.2	25.6	67.2	26.2	−2.57	−0.17	0.0103	No
Pasta								
Overall liking	50.4	27.1	62.9 *	26.4	−3.26	−0.22	0.0011	**Yes**
WTP	56.7	26.3	64.4 *	25.5	−3.26	−0.22	0.0011	**Yes**
Tastiness	55.3	25.5	62.5 *	24.5	−3.13	−0.21	0.0017	**Yes**
Quality	58.3	24.9	62.2	24.3	−2.05	−0.14	0.0407	No
Powerfulness	49.9	25.1	54.2	26.6	−1.94	−0.13	0.0523	No
Heaviness	53.0	26.2	52.1	25.9	−0.81	−0.05	0.4166	No
Attractiveness	45.5	25.9	56.0 *	30.4	−3.57	−0.24	0.0004	**Yes**
Lemon mousse								
Overall liking	49.7	27.6	61.1	26.6	−3.00	−0.20	0.0027	No
WTP	54.6	27.6	62.8	26.1	−2.81	−0.19	0.0050	No
Tastiness	63.3	26.8	68.3	24.2	−1.91	−0.13	0.0557	No
Quality	61.4	25.1	66.6	24.3	−2.15	−0.14	0.0317	No
Powerfulness	56.1	25.1	60.1	24.8	−1.10	−0.07	0.2705	No
Heaviness	47.8	25.8	47.1	26.4	−0.36	−0.02	0.7155	No
Attractiveness	56.6	26.3	63.8	25.3	−2.33	−0.16	0.0198	No

*r* = effect size (see Pallant, 2007, p. 225 [43]); Sig. = window positions where scores are significantly different using the *p*-value derived using the Hochberg procedure; the score that is significantly higher is marked with an asterisk and is presented in bold (*). WTP = Willingness to purchase. Note: Sample size = 110. *p*-values are rounded and shown to four decimal places due to the number of comparisons made.

## Appendix A

**Table A1:** Effect sizes for the ‘quarter-window’ (top-left/top-right/bottom-left/bottom-right) Wilcoxon signed-ranks tests by category.

	Top-Right (b) vs. Top-Left (a)	Top-Right (b) vs. Bottom-Left (c)	Bottom-Right (d) vs. Top-Left (a)	Bottom-Right (d) vs. Bottom-Left (c)
Granola				
Overall liking	0.33	-	0.39	0.50
WTP	0.32	-	0.39	0.41
Tastiness	0.35	-	0.38	0.39
Quality	-	-	0.47	0.50
Powerfulness	0.55	-	0.45	0.48
Heaviness	-	-	-	-
Attractiveness	0.50	-	0.50	0.45
Chocolates				
Overall liking	-	0.52	-	0.72
WTP	0.68	0.55	0.61	0.74
Tastiness	0.53	0.40	0.45	0.43
Quality	0.46	-	0.51	0.52
Powerfulness	0.54	-	0.45	0.53
Heaviness	-	-	-	-
Attractiveness	0.52	-	0.50	0.57
Pasta				
Overall liking	-	-	-	0.60
WTP	-	-	-	0.42
Tastiness	0.43	-	0.55	0.52
Quality	0.36	-	0.41	0.67
Powerfulness	-	-	-	0.44
Heaviness	-	-	-	-
Attractiveness	0.32	-	0.45	0.59
Lemon mousse				
Overall liking	-	-	-	-
WTP	0.57	0.46	-	0.36
Tastiness	0.46	-	0.39	-
Quality	0.62	-	0.50	0.37
Powerfulness	0.58	0.40	-	0.47
Heaviness	-	-	-	-
Attractiveness	0.59	0.40	0.48	0.39

Note: Standardised effect sizes (dz) are only shown where a significant difference was found. All significant differences shown were derived using the Hochberg procedure.
